# Supplementary material for: Intracerebral mechanisms explaining the impact of incidental feedback on mood state and risky choice
Source: eLife. 2022 Jul 13;11:e72440. doi: 10.7554/eLife.72440 (PMC9348847; doi:10.7554/eLife.72440)
Supplement: Supplementary file 1. [file elife-72440-supp1.docx]

# Supplementary file 1 - Tables of demographic data and statistical results

**Table S1**: **Demographic data**. M: male; F: female; L: left; R: right; A: ambidextrous; vmPFC: ventromedial prefrontal cortex; daIns: dorsal anterior insula

| ID | Sex | Age (years) | Epilepsy onset (age) | Suspected epileptic focus | Hand laterality | Number of electrodes | Number of recording sites | Number of recorded bipoles | Number of bipoles in vmPFC | Number of bipoles in daIns |
| --- | --- | --- | --- | --- | --- | --- | --- | --- | --- | --- |
| G1 | M | 46 | 23 | Right insulo-opercular / Left opercular | L | 17 | 122 | 85 | 0 | 2 |
| G2 | M | 38 | 15 | Left precentral / Premotor | L | 17 | 122 | 88 | 0 | 1 |
| G3 | M | 43 | 7 | Right temporal | R | 17 | 122 | 83 | 6 | 3 |
| G4 | F | 38 | 3 | Bilateral extensive | R | 18 | 122 | 77 | 0 | 2 |
| G5 | F | 35 | 4 | Several territories | R | 16 | 122 | 92 | 6 | 1 |
| G6 | F | 45 | 10 | Bi-temporal / Amygdala nucleus | R | 17 | 122 | 84 | 6 | 6 |
| G7 | F | 46 | 41 | Right temporal | R | 13 | 122 | 100 | 4 | 2 |
| G8 | F | 43 | 2 | Right insulo-opercular | R | 16 | 122 | 92 | 0 | 3 |
| G9 | M | 45 | 41 | Left mesio-temporal | R | 17 | 122 | 90 | 2 | 4 |
| L1 | F | 33 | 7 | Left insulo-opercular / Left amygdala | R | 13 | 137 | 123 | 2 | 4 |
| L2 | M | 56 | 36 | Left mesio-temporal | R | 13 | 141 | 127 | 2 | 3 |
| L3 | F | 38 | 27 | Right mesio-temporal | R | 9 | 89 | 79 | 2 | 0 |
| M1 | M | 56 | 35 | Left temporal | R | 14 | 183 | 168 | 0 | 3 |
| M2 | M | 34 | 4 | Temporo-frontal bilateral | R | 20 | 253 | 228 | 0 | 13 |
| N1 | M | 42 | 6 | Left fronto-opercular | R | 11 | 106 | 93 | 0 | 2 |
| P1 | M | 23 | 15 | Right frontal | R | 14 | 212 | 86 | 9 | 4 |
| P2 | M | 33 | 16 | Left temporal | R | 15 | 126 | 93 | 0 | 1 |
| P3 | F | 46 | 28 | Right temporal | R | 12 | 151 | 136 | 2 | 6 |
| R1 | M | 45 | 10 | Bilateral extensive | R | 13 | 126 | 112 | 3 | 2 |
| R2 | F | 21 | 2 | Right cingulate gyrus | R | 13 | 166 | 152 | 7 | 3 |
| R3 | M | 39 | 30 | Right temporal | R | 13 | 183 | 169 | 6 | 4 |
| R4 | F | 23 | 20 | Right temporo-insulo-perisylvian | A | 11 | 125 | 113 | 0 | 3 |
| R5 | F | 47 | 28 | Hippocampal sclerosis / Right temporal | R | 10 | 107 | 96 | 0 | 2 |
| R6 | M | 17 | 9 | Left temporo-insulo-frontal multifocal | R | 14 | 174 | 159 | 4 | 2 |
| R7 | F | 39 | 8 | Upper posterior frontal gyrus | R | 11 | 126 | 114 | 4 | 2 |
| R8 | M | 46 | 33 | Orbitofrontal / Right anterior temporal | R | 15 | 195 | 179 | 6 | 1 |
| R9 | F | 39 | 26 | Right medial temporal | R | 11 | 124 | 112 | 4 | 2 |
| R10 | M | 21 | 6 | Limbic cingulate | A | 13 | 168 | 154 | 6 | 3 |
| T1 | F | 49 | 33 | Right anterior temporal | R | 13 | 122 | 106 | 5 | 2 |
| T2 | M | 58 | 40 | Left fronto-temporal / Left fronto-mesial | R | 14 | 127 | 104 | 5 | 0 |

**Table S2**: **Association between mood levels and Broadband Gamma Activity (50-150 Hz)**. Areas are ordered according to the absolute t-value obtained with the linear mixed effect model. Blue: areas positively associated with both mood rating and TML; Red: areas negatively associated with both mood rating and TML; p-values are obtained with two-sided one-sample t-tests cluster-wise corrected (p_corr_ < 0.05).

|  | **Positive correlation with mood ratings** | | | | | |  |  | **Positive correlation with TML** | | | | | |
| --- | --- | --- | --- | --- | --- | --- | --- | --- | --- | --- | --- | --- | --- | --- |
| **ROI** | **Best cluster onset (s)** | **Best cluster offset (s)** | **Sum t-value** | **p-value** | **Linear mixed effect model** | |  | **ROI** | **Best cluster onset (s)** | **Best cluster offset (s)** | **Sum t-value** | **p-value** | **Linear mixed effect model** | |
|  |  |  |  |  | **t-value** | **p-value** |  |  |  |  |  |  | **t-value** | **p-value** |
| **vmPFC** | **-1,37** | **-1,04** | **122,26** | **0,010** | **3,00** | **3.10^-3^** |  | PCC | -3,63 | -3,26 | 123,76 | 0,025 | 3,19 | 2.10^-3^ |
| Hippocampus | -0,57 | -0,26 | 108,16 | 0,019 | 2,55 | 0,011 |  | **vmPFC** | **-0,57** | **-0,13** | **132,44** | **8.10**^-3^ | **3,00** | **3.10^-3^** |
|  |  |  |  |  |  |  |  | PMdl | -1,86 | -1,10 | 209,73 | 8.10^-4^ | 2,69 | 7.10^-3^ |
|  |  |  |  |  |  |  |  | PFrvl | -0,96 | -0,39 | 178,02 | 5.10^-3^ | 2,16 | 0,031 |

*Continued on next page*

|  | **Negative correlation with mood ratings** | | | | | |  |  | **Negative correlation with TML** | | | | | |
| --- | --- | --- | --- | --- | --- | --- | --- | --- | --- | --- | --- | --- | --- | --- |
| **ROI** | **Best cluster onset (s)** | **Best cluster offset (s)** | **Sum t-value** | **p-value** | **Linear mixed effect model** | |  | **ROI** | **Best cluster onset (s)** | **Best cluster offset (s)** | **Sum t-value** | **p-value** | **Linear mixed effect model** | |
|  |  |  |  |  | **t-value** | **p-value** |  |  |  |  |  |  | **t-value** | **p-value** |
| **daIns** | **-3,36** | **-2,51** | **-325,84** | **< 1.7.10^-5^** | **-5,24** | **2.10^-7^** |  | PMdm | -3,51 | -2,75 | -315,68 | 3.10^-5^ | -5,46 | 5.10^-8^ |
| Mdl | -2,82 | -2,43 | -144,57 | 6.10^-3^ | -4,74 | 2.10^-6^ |  | PFrm | -0,96 | -0,66 | -130,13 | 8.10^-3^ | -4,61 | 4.10^-6^ |
| PMdm | -1,78 | -1,39 | -142,83 | 4.10^-3^ | -4,65 | 3.10^-6^ |  | Sdm | -3,04 | -1,52 | -511,91 | < 1.7.10^-5^ | -4,12 | 4.10^-5^ |
| PMdl | -2,83 | -1,86 | -270,21 | 8.10^-5^ | -4,53 | 6.10^-6^ |  | Putamen | -3,03 | -2,65 | -144,64 | 5.10^-3^ | -4,08 | 5.10^-5^ |
| PFrd | -3,13 | -2,88 | -90,40 | 0,036 | -4,03 | 6.10^-5^ |  | Mdm | -4,00 | 0,00 | -2182,56 | < 1.7.10^-5^ | -3,42 | 6.10^-4^ |
| VCrm | -3,69 | -3,32 | -115,37 | 0,022 | -3,51 | 5.10^-4^ |  | VCrm | -0,28 | 0,00 | -118,65 | 0,025 | -2,98 | 3.10^-3^ |
| SPC | -2,34 | -1,97 | -146,23 | 0,016 | -3,36 | 8.10^-4^ |  | Mv | -4,00 | -3,66 | -98,30 | 0,037 | -2,84 | 5.10^-3^ |
| MCC | -1,01 | -0,70 | -93,00 | 0,038 | -3,07 | 2.10^-3^ |  | VCl | -0,72 | -0,31 | -140,68 | 0,010 | -2,64 | 8.10^-3^ |
| Mv | -1,48 | -0,91 | -176,84 | 2.10^-3^ | -3,05 | 2.10^-3^ |  | ITCm | -0,89 | 0,00 | -342,32 | 2.10^-5^ | -2,59 | 0,010 |
| PMrv | -2,59 | -1,85 | -261,59 | 7.10^-5^ | -3,00 | 3.10^-3^ |  | **daIns** | **-3,13** | **-2,72** | **-136,38** | **9.10^-3^** | **-2,51** | **0,012** |
| IPCd | -2,49 | -2,01 | -142,36 | 0,011 | -2,91 | 4.10^-3^ |  | Pfrdls | -2,43 | -2,11 | -110,52 | 0,019 | -2,24 | 0,025 |
| Sdl | -3,28 | -2,83 | -155,98 | 9.10^-3^ | -2,84 | 5.10^-3^ |  |  |  |  |  |  |  |  |
| Pfrdls | -3,31 | -2,77 | -160,08 | 2.10^-3^ | -2,72 | 7.10^-3^ |  |  |  |  |  |  |  |  |
| VCl | -0,76 | -0,41 | -114,61 | 0,016 | -2,68 | 7.10^-3^ |  |  |  |  |  |  |  |  |
| IPCv | -2,82 | -2,16 | -214,19 | 3.10^-4^ | -2,53 | 0,012 |  |  |  |  |  |  |  |  |
| OFCvl | -3,21 | -2,87 | -90,67 | 0,042 | -2,49 | 0,013 |  |  |  |  |  |  |  |  |
| OFCvm | -0,93 | -0,46 | -128,83 | 9.10^-3^ | -2,33 | 0,020 |  |  |  |  |  |  |  |  |

**Table S3**: **Association between choice model residues and Broadband Gamma Activity (50-150 Hz)**. Areas are ordered according to the absolute t-value obtained with the linear mixed effect model; p-values are obtained with two-sided one-sample t-tests cluster-wise corrected (p_corr_ < 0.05).

|  | **Positive correlation with residual error of choice** | | | | | |  |  | **Negative correlation with residual error of choice** | | | | | |
| --- | --- | --- | --- | --- | --- | --- | --- | --- | --- | --- | --- | --- | --- | --- |
| **ROI** | **Best cluster onset (s)** | **Best cluster offset (s)** | **Sum t-value** | **p-value** | **Linear mixed effect model** | |  | **ROI** | **Best cluster onset (s)** | **Best cluster offset (s)** | **Sum t-value** | **p-value** | **Linear mixed effect model** | |
|  |  |  |  |  | **t-value** | **p-value** |  |  |  |  |  |  | **t-value** | **p-value** |
| MCC | -0,44 | -0,10 | 104,25 | 0,013 | 3,91 | 9.10^-5^ |  | PMrv | -0,84 | -0,45 | -128,62 | 7.10^-3^ | -3,33 | 9.10^-4^ |
| Mdl | -1,41 | -0,73 | 227,03 | 3.10^-4^ | 2,84 | 5.10^-3^ |  | pINS | -1,32 | -0,93 | -135,50 | 3.10^-3^ | -2,89 | 4.10^-3^ |
| PFcdm | -1,83 | -1,51 | 84,25 | 0,033 | 2,75 | 6.10^-3^ |  | IPCv | -1,69 | -1,32 | -107,48 | 0,013 | -2,89 | 4.10^-3^ |
| VCl | -0,46 | 0,00 | 175,98 | 1.10^-3^ | 2,40 | 0,016 |  | SPC | -1,80 | -1,16 | -235,08 | 1.10^-3^ | -2,58 | 0,010 |
| **vmPFC** | **-1,64** | **-1,31** | **91,20** | **0,020** | **2,29** | **0,022** |  | **daIns** | **-0,95** | **-0,67** | **-85,17** | **0,029** | **-2,04** | **0,041** |
| ITCr | -0,31 | -0,02 | 72,76 | 0,040 | 2,19 | 0,029 |  |  |  |  |  |  |  |  |
| vaINS | -2,00 | -1,54 | 111,89 | 9.10^-3^ | 2,09 | 0,036 |  |  |  |  |  |  |  |  |
| PFrm | -0,33 | 0,00 | 99,46 | 0,016 | 2,06 | 0,039 |  |  |  |  |  |  |  |  |
| STCc | -0,49 | 0,00 | 161,28 | 2.10^-3^ | 1,98 | 0,047 |  |  |  |  |  |  |  |  |

**Table S4**: Associations between BGA (in the vmPFC or daIns) and mood rating, theoretical mood level (TML) or risky choice, using statistics across subjects (group-level random-effects analyses).

| **BGA and MOOD** | | | | |
| --- | --- | --- | --- | --- |
| **Parcel** | **Mean regression coef** | **t-value** | **df** | **p-value** |
| vmPFC | 0,04 | 2,09 | 19 | 0,050 |
| daIns | -0,05 | -3,89 | 27 | 6.10^-4^ |

| **BGA and TML** | | | | |
| --- | --- | --- | --- | --- |
| **Parcel** | **Mean regression coef** | **t-value** | **df** | **p-value** |
| vmPFC | 0,02 | 2,13 | 19 | 0,047 |
| daIns | -0,02 | -2,11 | 27 | 0,045 |

| **BGA and CHOICE** | | | | |
| --- | --- | --- | --- | --- |
| **Parcel** | **Mean regression coef** | **t-value** | **df** | **p-value** |
| vmPFC | 0,08 | 2,44 | 19 | 0,024 |
| daIns | -0,05 | -2,19 | 27 | 0,037 |
